# Presence of Immune Complexes of IgG/IgM Bound to B2-glycoprotein I Is Associated With Non-criteria Clinical Manifestations in Patients With Antiphospholipid Syndrome

**DOI:** 10.3389/fimmu.2018.02644

**Published:** 2018-11-20

**Authors:** Dolores Pérez, Ljudmila Stojanovich, Laura Naranjo, Natasa Stanisavljevic, Gordana Bogdanovic, Manuel Serrano, Antonio Serrano

**Affiliations:** ^1^Immunology Department, Hospital 12 de Octubre, Madrid, Spain; ^2^Internal Medicine, “Bezanijska Kosa”, University Medical Center, Belgrade, Serbia

**Keywords:** antiphospholipid, livedo reticularis, sicca, thrombocytopenia, leukopenia, circulating immune-complexes, anti-beta-2-glycoprotein I, non-criteria APS clinical manifestations

## Abstract

**Background:** Antiphospholipid syndrome (APS) is an acquired autoimmune disorder defined by the presence of both clinical (thromboembolic events or pregnancy morbidity) and laboratory (antiphospholipid antibodies, aPL) manifestations. Despite their importance, several clinical manifestations strongly associated with APS such as livedo reticularis (LR), thrombocytopenia, sicca-ophthalmic(sicca), heart, or neurological manifestations are not included in the APS clinical classification criteria. Circulating immune complexes (CIC) formed by Beta-2-glycoprotein I (B2GPI) and aPL (B2-CIC) have been described and their presence has been related with thrombotic events.

**Methods:** Cross-sectional and observational cohort study in APS patients with thrombotic symptomatology.

**Setting and Participants:** Fifty-seven patients from the University Hospital Center Bezanijska Kosa (Belgrade, Serbia) who met the APS classification criteria (35 with primary APS and 22 with APS associated to systemic lupus erythematosus). This study aimed to determine the prevalence of B2-CIC in APS patients and to evaluate their association with clinical manifestations of APS not included in the classification criteria.

**Results:** B2-CIC prevalence in APS patients was 19.3%. The presence of thrombocytopenia (OR:5.7), livedo reticularis (OR:5.6), sicca (OR:12.6), and leukopenia (OR:5.6) was significantly higher in patients with B2-CIC than in the rest of APS patients. C3 and C4 complement factor levels were significantly lower in B2-CIC positive patients, which suggests a greater consumption of complement. Patients with quadruple aPL positivity (triple aPL-positivity plus the presence of B2-CIC) showed a higher prevalence of thrombocytopenia, leucopenia and LR than those with single/double aPL-positivity. No significant differences were found in the frequencies observed in patients with triple-only vs. single/double aPL-positivity. There were no significant differences between patients with primary APS and lupus-associated APS regarding the prevalence of B2-CIC and outcomes.

**Conclusions:** Presence of B2-CIC is strongly associated with several non-criteria clinical manifestations related to APS and to higher complement consumption. More studies are required to better understand the clinical significance of B2-CIC.

## Key messages

- Prevalence of B2-CIC (IgG or IgM isotypes) in APS patients is 19.29%. - B2-CIC are strongly associated with livedo reticularis and ophthalmic sicca.
- Thrombocytopenia, leukopenia and complement consumption are related with presence of B2-CIC.
- The quadruple aPL positivity (triple aPL positivity in the presence of B2-CIC) is highly associated with ophthalmic sicca and thrombocytopenia.

## Introduction

Antiphospholipid Syndrome (APS) is an autoimmune disorder characterized by thrombosis and/or pregnancy morbidity in presence of persistent antiphospholipid antibodies such as lupus anticoagulant (LA), isotype IgG and/or IgM for anti-beta-2-glycoprotein I (aB2GPI) and anti-cardiolipin antibodies (aCL) (1–4).

The current APS international classification criteria require the presence of at least one laboratory criterion on two or more occasions, at least 12 weeks apart as well as the presence of a minimum of one clinical criterion (5). APS could manifest as primary (without other concomitant morbidity), as associated to other autoimmune disease, mainly Systemic lupus erythematosus (SLE) (6) or as catastrophic-APS (multiple organ thrombosis with a high mortality rate) (7).

The APS clinical spectrum goes far beyond the classification criteria (8). There are other clinical manifestations associated with the syndrome such as heart valve disease (9), neurological manifestations (headache, migraine, or epilepsy) (10), livedo reticularis (LR) (11), thrombocytopenia (12), and nephropathy (13). Several studies have supported the strong association between APS and these clinical manifestations. However, these manifestations have not been included as clinical criteria because of their low specificity (14, 15). Similarly, other aPL antibodies as aB2GPI of IgA isotype (16), anti-Annexin A2 and A5 (17), anti-vimentin and anti-phosphatidilserin/prothrombin (18) have been associated with APS but also have not been included as laboratory criteria because there is still not sufficient evidence to include them (14).

Currently, several authors have been emphasizing the need for new APS biomarkers to improve sensitivity and specificity in the diagnosis of the syndrome (19, 20). Presence of immune complexes of IgA aB2GPI antibodies bound to B2GPI (B2A-CIC) has been described recently in the blood of patients with clinical thrombotic manifestations for APS (21, 22). The prevalence of B2A-CIC has been found to be significantly higher in patients with acute thrombotic event (22). The risk of developing thrombosis immediately after undergoing transplant surgery is significantly higher (HR: 6.72) in patients with B2A-CIC (23). Transplant surgery is a known second hit for the triggering of an APS event (24).

The immune complexes of aPL have been described previously (25) and they have recently been visualized with high resolution microscopy (26). Presence of immune complexes was associated with the occurrence of acute thrombotic events (27–29). However, prevalence in APS patients of circulating immune complexes between aPL and B2GPI (B2-CIC) and the relationship of these complexes with APS-associated clinical manifestations have still not been described.

This study has aimed to determine the prevalence of B2-CIC of IgG and/or IgM isotypes (B2G-CIC and B2M-CIC, respectively) in patients with clinical and laboratory classification criteria of APS patients and the relationship between presence of these biomarkers and the APS-related clinical manifestations.

## Patients and methods

### Study design

A cross-sectional study developed to determine the prevalence of B2-CIC in APS patients and their association with the APS-related clinical manifestations of these subjects.

### Patients

A total of 57 patients with a diagnosis of thrombotic APS who met revised criteria for APS (5) were consecutively enrolled in the year 2000 in the University Hospital Center Bezanijska Kosa. All patients were examined by a combined group of rheumatologist, neurologist, cardiologist, radiologist, hematologist, and ophthalmologist. Of these, 35 had PAPS while 22 had APS in association with SLE. All the patients had thrombosis and some had gestational morbidity consistent with APS in accordance with the 2006 revised criteria for APS (5). Mean age was 47.6 ± 1.6 years; 36 (63.2%) were women. Main population characteristics are shown in Table 1 (additional data are shown in Supplementary Table 1). Subjects were divided into two groups according to positivity for B2-CIC (group-1) and negativity for CIC (group-2). Diagnosis of APS was made by presence of aPL and other diagnostic criteria (Doppler ultrasound, computed tomography, heart ultrasound or others for arterial and/or venous thrombosis, and multiple and recurrent fetal losses). All SLE-diagnosed patients met the American College of Rheumatology (ACR) classification criteria (30). Disease activity was assessed at the time of enrolment in the study using the Systemic Lupus Erythematosus Disease Activity Index (SLEDAI) score, and only those patients with stable disease were enrolled (31). Clinical data and medication were obtained from the patient's clinical database and records. Exclusion criteria included acute or chronic infection, marked renal and liver impairment or data of present or treated malignancy. The presence of comorbidities, such as arterial systemic hypertension (blood pressure ≥ 140 x 90 mmHg or use of anti-hypertensive drugs) and diabetes (glycated hemoglobin 7% or use of medication) and data on smoking habit (any person who smokes every day, occasional smoking and having quit smoking < 1 year ago) and drugs used were also evaluated. Four of the patients showed the presence of catastrophic APS (CAPS) and were included in the international registry of catastrophic APS patients (CAPS Registry) created in 2000 by the European Forum on Antiphospholipid Antibodies.

**Table 1:** Population description and clinical characteristics of the 57 APS patients included in the study.

**Condition**	***N* = 57 Mean/Number of patients**	**% / SEM**
Age (years)	47.6	±1.6
Sex (women)	36	(63.2%)
Catastrophic APS	4	(7.0%)
Primary APS	35	(61.4%)
Disease duration (years)	5.4	±0.7
Diabetes Mellitus	2	(3.5%)
Hypertension	5	(8.8%)
Dyslipidemia	5	(8.8%)
Smoker	21	(36.8%)
**ANTIPHOSPHOLIPID ANTIBODIES (APL) POSITIVE**		
Anti-cardiolipin IgG antibodies	24	(42.1%)
Anti-cardiolipin IgM antibodies	25	(43.9%)
Anti-beta2-glycoprotein I IgG antibodies	26	(45.6%)
Anti-beta2-glycoprotein I IgM antibodies	29	(50.9%)
Lupus anticoagulant	38	(66.7%)
Triple aPL positivity	15	(26.3%)
**APS PATHOLOGY**		
***Gestational morbidity***		
Women in fertile age	34	(59.6%)
Women with fetal loss	21	(61.8%)
Mean fetal loss	1.6	±0.2
Women with late fetal loss	15	(44.1%)
Women with early fetal loss	6	(17.6%)
***Thrombotic events***		
Arterial thrombosis	44	(77.2%)
Venous thrombosis	27	(47.4%)
Inferior extremity deep vein thrombosis	12	(21.1%)
Superior extremity arterial thrombosis	3	(5.3%)
Pulmonary embolism	14	(24.6%)

*SEM, standard error of the mean. Early fetal loss: miscarriage during the first 13 weeks, the first trimester, Late fetal loss: miscarriage after the first 13 weeks of gestation*.

### Ethical issues

The “Belgrade APS cohort” observational and non-interventional study was approved by the Ethics Committees of the Bezanijska Kosa, University Medical Center of Belgrade. Informed consent was obtained from all individual participants included in the study.

After blood sampling, a blind code was assigned to serum of each patient to assure anonymity throughout the process. The blind numerical code associate each serum with the corresponding clinical data. The sera that were sent to the Spanish group did not contain DNA and, so according to Spanish legislation, were not considered as human biological samples that require ethical authorization for their use (article 3 of the Spanish Biomedical Research Law of 2007).

As it is a non-interventionist observational study initiated in Belgrade, the clinical data were anonimized, the sera were obtained before 2007 and do not contain information about the characteristic genetic endowment of a person (DNA), Spanish legislation does not require special ethical treatment or additional informed consent for this type of study.

### Definitions

*Thrombotic event:* thrombosis in any arterial or venous blood vessel. The diagnosis was confirmed using objective validated criteria such as imaging techniques (5).

*Gestational Morbidity*: deaths of a morphological normal fetus, premature births or spontaneous abortions defined in accordance with the International Consensus Statement for Antiphospholipid Syndrome (5).

*Leukopenia*: number of blood white cells < 4,000 per microliter of blood.

*Thrombocytopenia*: number of platelets < 140,000 per microliter of blood.

*Ophthalmic sicca (sicca):* multifactorial disease characterized by insufficient tear production resulting in instability of the tear film, discomfort, sensation of visual dryness and visual disturbance. The patients with sicca were positive for Schirmer's test, performed without anesthesia (<5 mm in 5 min) and Rose Bengal score or another ocular dye score (>4 according to van Bijsterveld's scoring system).

*Triple positivity of aPL:* patients who are positive for the three laboratory markers associated with APS: LA, aCL and aB2GPI antibodies (IgG or/and IgM isotypes) (32).

Quadruple positivity of aPL or aPL “poker”: patients with triple positivity of aPL who also are positive for B2-CIC.

#### Triple only aPL positivity: patients with aPL triple positivity who are B2-CIC negative

*Systemic lupus erythematosus (SLE):* Diagnosis of SLE is based upon ACR classification criteria (31). The proposed classification is based on 11 criteria. Person shall be said to have systemic lupus erythematosus if any 4 or more of the 11 criteria are present, serially or simultaneously, during any interval of observation.

### Laboratory determinations

LA, aCL, and aB2GPI antibodies (IgG and IgM) were measured using the plasma (LA) and serum samples (aCL and aB2GPI) obtained at the moment of the patient's enrolment in the study, this being more than 3 months after the APS event. The serum samples were stored frozen at −30°C.

LA was based on the use of two different screening tests: diluted activated partial thromboplastin time and sensitive activated partial thromboplastin time according to the International Society on Thrombosis and Hemostasis (ISHT) recommendations (33). LA tests were performed while the patients were not receiving anticoagulant therapy.

IgG/IgM aCL and aBGPI antibodies were measured by an enzyme-linked immunosorbent assay (Binding Site Group Ltd, Birmingham, UK). Antibodies aCL levels were expressed in GPL or MPL phospholipids units (GPL-U and MPL-U). Antibodies aB2GPI levels were expressed in U/ml.

Positivity of aCL and anti-B2GPI of IgG and IgM isotypes was confirmed by BioPlex 2200 multiplex immunoassay system using BioPlex 2200 APLS IgG and APLS IgM panels (Bio-Rad, Hercules CA, USA). Antibody levels higher than 20 U/mL were considered positive following the manufacturer's guidelines, this coinciding with the 99th percentile of the European population determined in the laboratory.

Antibodies Against dsDNA, Chromatin, SSA-52 kDa (Ro52), SSA-60 kDa (Ro60), SSB (La), Sm, Sm/RNP, RNP-A, RNP-68 kDa, Scl70, Centromere B, Jo-1, And P Ribosomal Proteins Were Evaluated by BioPlex 2200 BioPlex® 2200 ANA Screen Panel (Bio-Rad, Hercules CA, USA).

Complement factors C3 and C4 levels were measured using Beckman Coulter IMMAGE Immunochemistry System (Beckman Coulter Inc. Pasadena, CA, USA). The range of normality for C3 levels was 88–225 mg/dL and for C4 levels 12–75 mg/dL.

Quantification of B2G-CIC and B2M-CIC levels was performed as previously described (21). Briefly, 96 wells Nunc maxisorp™ plates (A/S Nunc, Kamstrup, Roskilde, Denmark) were coated overnight at 4°C with mouse monoclonal antibody anti-human B2GP1 H219 (Mabtech AB, Nacka Strand, Sweden) at 2 μg/mL in PBS pH 7.4. Plates were washed (PBS 0.1% tween 20), blocked with PBS containing 1% bovine serum albumin (Sigma-Aldrich, St. Louis, MO, USA) 30 min at room temperature (RT) and washed (PBS 0.1% tween 20). Serum diluted at 1:100 in PBS were dispensed (100 μL/well; duplicates) and incubated 2 h at RT.

Anti-human IgG HRP-conjugate was used to detect B2G-CIC and anti-human IgM HRP-conjugate was used to detect B2M-CIC, (both from INOVA Diagnostics Inc., San Diego, CA, USA). The concentration of CIC-G or CIC-M (U/mL) of each serum was obtained by interpolating the mean optical density values with a calibration curve.

Sera with B2-CIC levels (IgG or IgM) higher than 21 AU were considered positive (99th percentile of healthy population). All the procedures were performed in a Triturus® Analyzer (Diagnostics Grifols, S.A. Barcelona, Spain).

### Statistical methods

Results were expressed as absolute frequency, percentage or mean ± standard error. The Pearson χ^2^ test (or Fisher's exact test, when appropriate) was used to determine the association between qualitative variables. Odds ratio (OR) was used to measure the strength of association between the presence of a risk factor and an outcome. OR and 95% confidence interval were calculated by logistic regression.

Student's *t-*test was used for comparisons in scaled variables with two previous categories of assessment of normality with Kolmogorov-Smirnov test. Mann-Whitney *U*-test was used for comparisons when the outcome was not normally distributed. Probabilities <0.05 were considered significant. A box-and-whisker plot represents the values from the lower to upper quartile (25 to 75 percentile) in the central box. The median is represented as the middle line in the box. Adjustment of *p*-values for multiple comparisons were obtained by the false discovery rate method (34).

Data were processed using Medcalc for Windows version 17.9 (Medcalc Software, Ostend, Belgium) and the “R” programming language (R Foundation for Statistical Computing, Vienna, Austria) (35).

## Results

### Prevalence of B2-CIC in APS patients

Eleven (19.3%) out of the 57 patients enrolled in the study had B2-CIC in the serum samples (group-1); 8 with B2M-CIC and 3 with B2G-CIC. None of the patients had both CIC formed. The remaining 46 patients had no CIC (group-2). The box and wisher data graph of B2M-CIC and B2G-CIC values is represented in Supplementary Figure 1.

No differences were observed in distribution of sex, age, proportion of women in fertile age and proportion of PAPS between both groups. The proportion of positivity for aPL was similar in individuals with and without CIC. No significant correlation was observed between levels of B2-CIC and aPL (not shown). LA was more frequent in patients in group-1 (90 vs. 60.9%,) but the difference was not statistically significant (*p* = 0.079). No significant differences were observed between both groups in the main APS-symptomatology (thrombotic events or gestational morbidity) presented by patients (Supplementary Table 2).

### APS-Related clinical manifestations

The prevalence of LR was significantly higher in individuals with B2-CIC in the serum sample than those without B2-CIC (63.6 vs. 23.9%, OR = 5.57, 95% CI: 1.37–22.65).

Patients with B2-CIC had a significantly higher incidence of sicca than group 2 (54.5 vs. 8.7%, OR: 12.6, 95% CI:2.63–60.48). All the patients were negative for rheumatoid factor (RF), anti-Ro60, anti-Ro52, and anti-La autoantibodies.

In patients with B2-CIC a significantly higher proportion of thrombocytopenia (54.5 vs. 17.4%, OR:5.7, 95% CI: 1.39–23.36) and leukopenia (45.5 vs. 13%, OR: 5.56, 95% CI: 1.28–24.03) was observed (Table 2).

**Table 2:** Extra-criteria clinical manifestations of antiphospholipid syndrome.

**Condition**	**Group 1**		**Group 2**				
	**B2-CIC + *N* = 11**	**%**	**B2-CIC – *N* = 46**	**% / SEM**	***P***	***P* adjusted**	**OR(95%CI)**
Valve thickening and dysfunction	0	(0%)	2	(4.3%)	0.481	0.561	
Livedo reticularis	7	(63.6%)	11	(23.9%)	0.011	0.025	5.57 (1.37-22.65)
Ophthalmic sicca	6	(54.5%)	4	(8.7%)	0.002	0.014	12.6 (2.63-60.48)
Thrombocytopenia	6	(54.5%)	8	(17.4%)	0.010	0.025	5.7 (1.39-23.36)
Autoimmune hemolytic anemia	1	(9.1%)	1	(2.2%)	0.351	0.491	
Leukopenia	5	(45.5%)	6	(13%)	0.014	0.025	5.56 (1.28-24.03)
Chorea	1	(9.1%)	2	(4.3%)	1.0	0.990	

*Group 1: patients with circulating immune complexes of IgG or IgM bound to beta-2-glycoprotein I (B2-CIC +). Group 2: patients without circulating immune-complexes (B2-CIC –). P-value < 0.05 was considered statistical significant, OR, odd ratio; CI, confidence interval. P adjusted: P-values adjusted for multiple comparisons*.

### Complement levels

**Figure 1:**
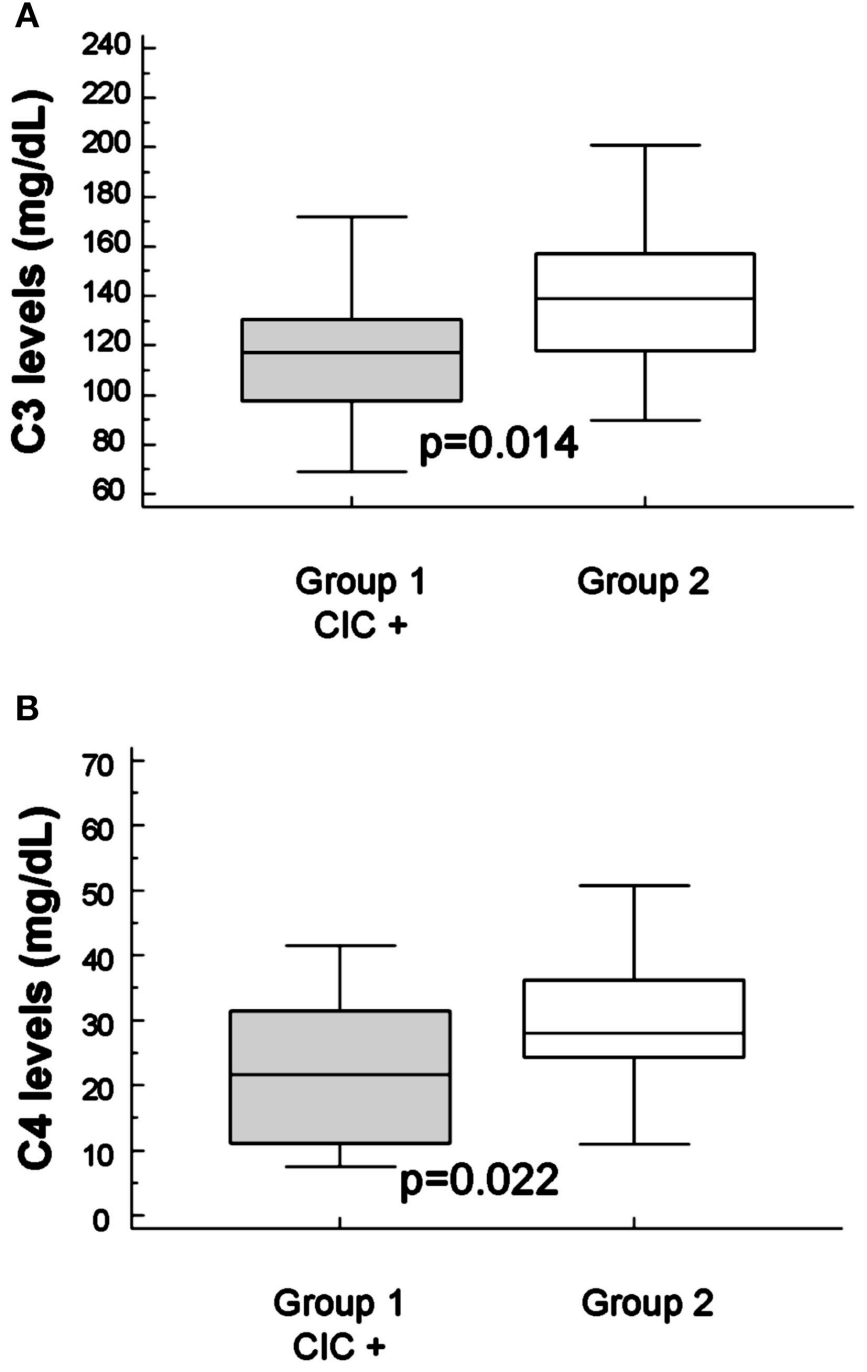
Mean levels of C3 **(A)** and C4 **(B)** complement in groups. Mean levels of C3 (115.6 ± 9.2 and 140.9 ± 4.3 mg/dL, group-1 and group-2, respectively) and mean levels of C4 (140.9 ± 4.3 and 30.8 ± 1.6 mg/dL, group-1 and group-2, respectively).

The mean levels of C3 and C4 complement factors were within the normal range in both groups. However, the C3 levels were significantly lower in group-1 than in group-2 (115.6 ± 9.2 vs. 140.9 ± 4.3 mg/dL; *p* = 0.014; Figure 1A). Also, C4 levels were decreased in patients with B2-CIC (22.0 ± 3.4 vs. 30.8 ± 1.6 mg/dL; *p* = 0.022; Figure 1B).

**Figure 2:**
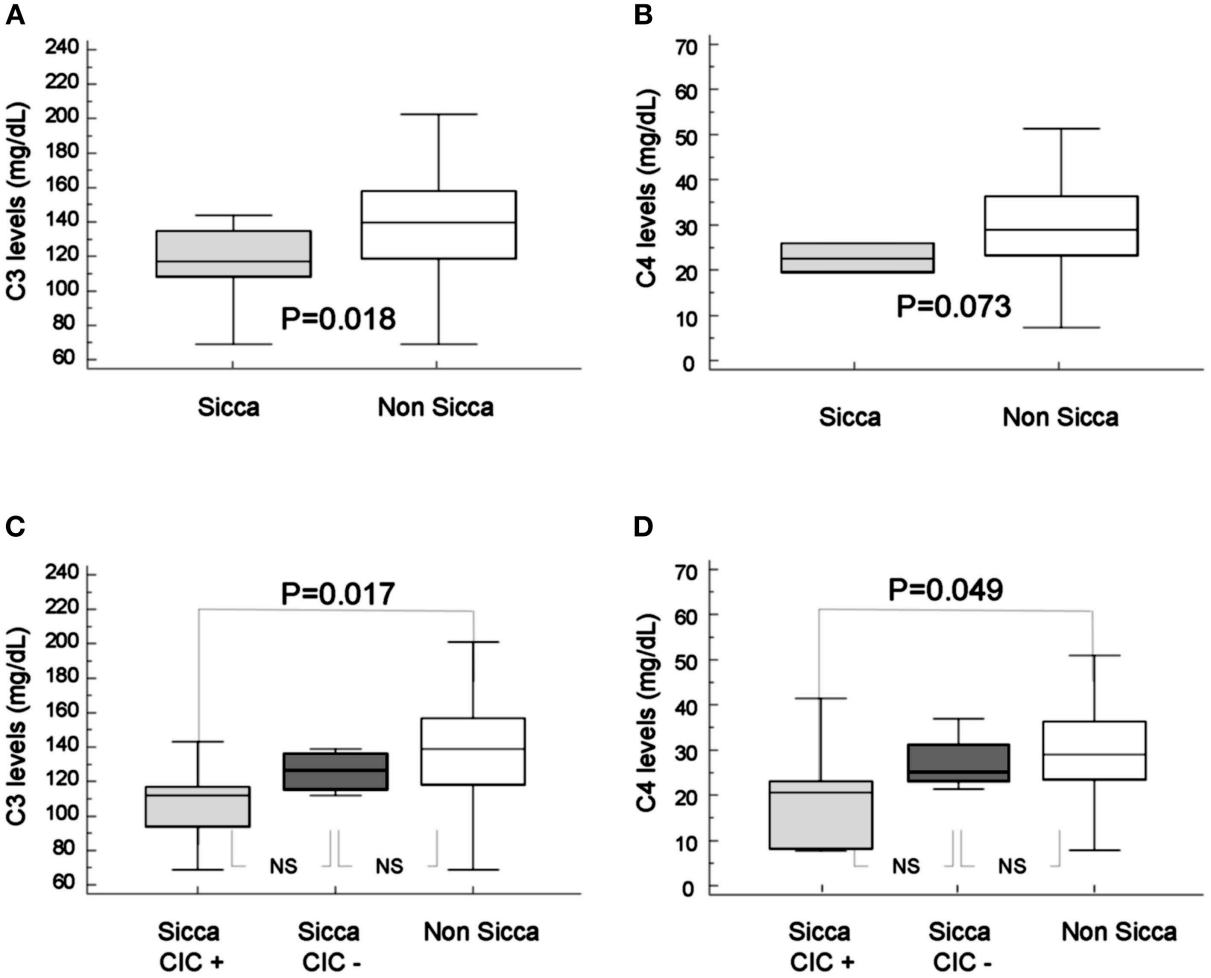
Mean levels of C3 **(A)** and C4 **(B)** complement in patients with ophthalmic sicca (gray box) and without sicca (white box). Mean levels of C3 **(C)** and C4 **(D)** complement in patients with sicca and circulating immune-complexes (gray box), with sicca and without circulating immune-complexes (dark box) and without sicca (white box). Mean levels of C3 and C4 in 10 patients with sicca (115.1 ± 6.9 and 23.1 ± 3.4 mg/dL, respectively) and mean levels of C3 and C4 in 47 patients without sicca (140.5 ± 4.5 and 30.4 ± 1.7 mg/dL, respectively). Levels of C3 were significantly lower in patients with sicca (*p* = 0.013), levels of C4 were lower but the difference was not statistically significant (*p* = 0.073).

The levels of C3 factor in patients with sicca was significantly lower than in the individuals without this clinical manifestation related to APS (115.1 ± 6.9 vs. 140.1 ± 4.5 mg/dL; *p* = 0.018, Figure 2A). Furthermore, C4 levels were lower in sicca (23.1 ± 3.4 vs. 30.4 ± 1.7) patients but the difference was not statistically significant (*p* = 0.073, Figure 2B).

Patients with sicca who were B2-CIC positive showed much lower C3 complement factors levels than patients without sicca (107.9 ± 24.8 vs. 140.5 ± 31.1 mg/dL; *p* = 0.013; *T*-test). C4 complement levels were also significantly lower in patients with sicca and B2-CIC (median: 20.6, interquartile range: 8.3–23.2 vs. median 28.7, interquartile range 23.1–36.0; *p* = 0.049). The C4 levels in patients with sicca and B2-CIC did not follow a normal distribution and they were analyzed with the Mann-Whitney test (Figures 2C,D).

### Triple and quadruple aPL positivity

Fifteen out of the 57 patients involved in the study were triple positive for aPL. The prevalences of pulmonary embolism (46.7 vs. 16.7%; *p* = 0.022) and catastrophic APS (20 vs. 2.4%; *p* = 0.023) were significantly higher in patients with triple aPL positivity than in individuals with single or double aPL positivity (Table 3). However, the remaining clinical manifestations did not show significant differences between both groups of patients (Table 3 and Supplementary Table 3).

**Table 3:** Clinical characteristics of APS patients with triple aPL positivity vs. single or double aPL positivity.

**Condition**	**Triple positivity *N* = 15**	**% / SEM**	**Single / double positivity *N* = 42**	**% / SEM**	***p*-value**
Age (years)	46	±3.0	48.2	±1.9	0.548
Sex (women)	11	(73.3%)	25	(59.5%)	0.341
Catastrophic APS	3	(20%)	1	(2.4%)	0.023
Primary APS	7	(46.7%)	28	(66.7%)	0.172
B2GPI Immune complexes (IgG or IgM)	4	(26.7%)	7	(16.7%)	0.400
B2GPI Immune complexes IgG	1	(6.7%)	2	(4.8%)	0.777
B2GPI Immune complexes IgM	3	(20%)	5	(11.9%)	0.439
**APS PATHOLOGY**					
***Gestational morbidity***					
Women in fertile age	9	(60%)	25	(59.5%)	0.974
Mean fetal loss	1.6	±0.3	1.6	±0.2	0.972
***Thrombotic events***					
Arterial thrombosis	13	(86.7%)	31	(73.8%)	0.308
Venous thrombosis	8	(53.3%)	19	(45.2%)	0.590
Pulmonary embolism	7	(46.7%)	7	(16.7%)	0.022
Inferior extremity deep vein thrombosis	3	(20%)	9	(21.4%)	0.907
Superior extremity arterial thrombosis	2	(13.3%)	1	(2.4%)	0.103
**VASCULAR RISK FACTORS**					
Diabetes Mellitus	0	(0%)	2	(4.8%)	1.000
Hypertension	1	(6.7%)	4	(9.5%)	0.737
Dyslipidemia	2	(13.3%)	3	(7.1%)	0.467
Trauma	1	(6.7%)	2	(4.8%)	0.777
Smoke	5	(33.3%)	16	(38.1%)	0.743
**OTHER IMMUNOLOGICAL MARKERS**					
Reactive protein C elevated	4	(26.7%)	9	(21.4%)	0.678
CH50	0	(0%)	1	(2.4%)	0.547
C3 complement factor low	0	(0%)	3	(7.1%)	0.288
C4 complement factor low	1	(6.7%)	0	(0%)	0.263
Anti-DNA antibodies	3	(20%)	3	(7.1%)	0.164
Antinuclear autoantibodies	5	(33.3%)	19	(45.2%)	0.423
Rheumatoid Factor	0	(0%)	2	(4.8%)	1.000
Anti-Ro antibodies	0	(0%)	2	(4.8%)	1.000
Anti-La antibodies	0	(0%)	1	(2.4%)	1.000

Four of the 15 patients with triple positivity were also positive for B2-CIC, a situation which was considered as “quadruple aPL positivity” or “aPL poker” (triple positivity plus the presence of B2-CIC). The 11 patients with triple positivity who were B2-CIC negative were considered as “triple-only” aPL positive.

**Figure 3:**
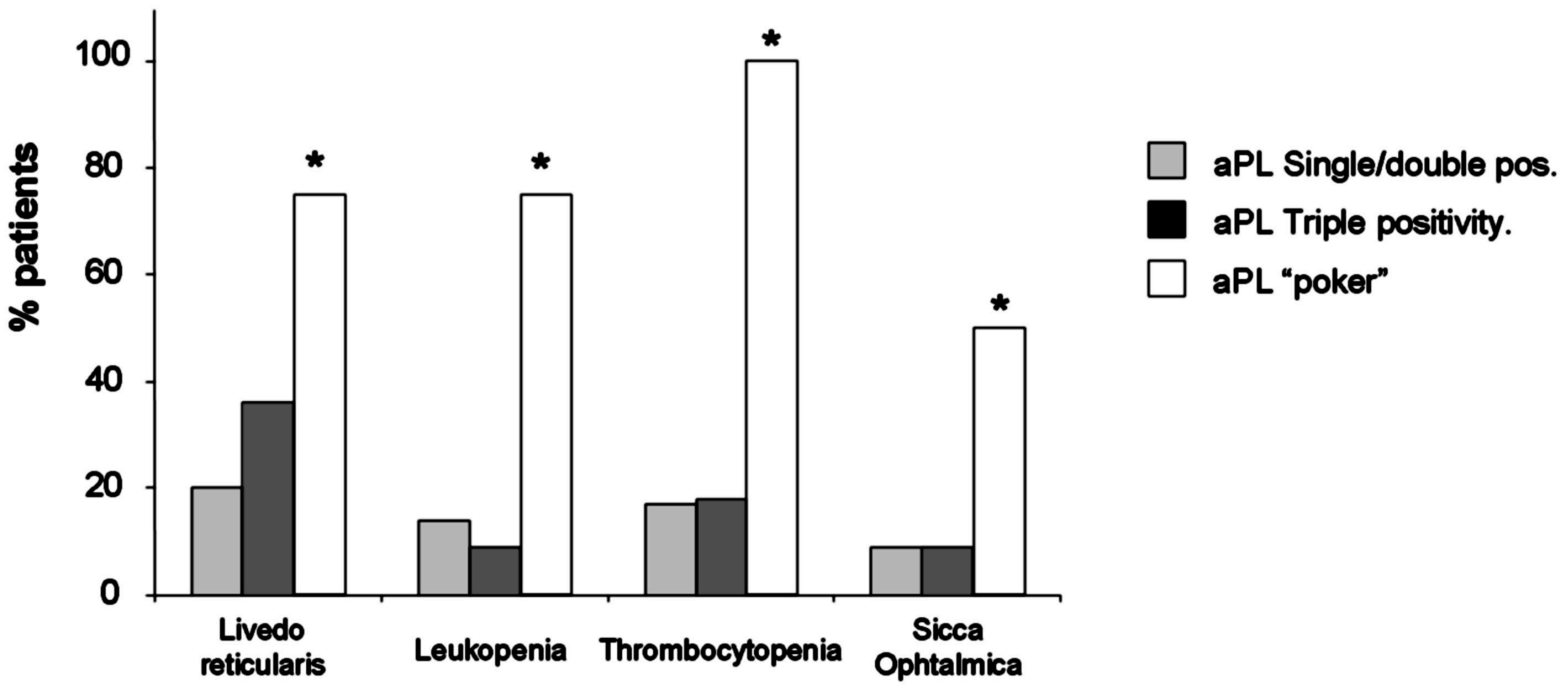
Prevalence of aPL simple/double positivity (gray bar), triple aPL positivity (dark bar), and quadruple aPL positivity or poker aPL positivity (white bar) in patients with livedo reticularis, ophthalmic sicca, leukopenia and thrombocytopenia. *p < 0.05 respect simple/double positivity.

Presence of LR, leucopenia and thrombocytopenia is significantly higher in patients with quadruple aPL positivity than in the patients with single or double aPL positivity (75, 75, 100 vs. 20, 14, 17; odds ratio: 12, 18, and 40.8; *p*-values: 0.043, 0.021, 0.018; Fisher's exact test). When the presence of these clinical characteristics was compared in patients with triple-only aPL positivity vs. those of single or double positivity, the differences were not significant (6, 9, 18 vs. 20, 14, 17; *p*-values: 0.274, 0.658, 0.937) (Figure 3 and Table 4).

**Table 4:** Clinical characteristics of APS patients with quadruple (left) and triple-only (right) aPL positivity vs. patients with single or double positivity.

**Condition**	**Quadruple aPL positivity**			**Triple-only aPL positivity**		
	**OR**	**95% CI**	***p***	**OR**	**95% CI**	***p***
Livedo reticularis	12	1.1 to 133.6	0.043	2.3	0.5 to 10.1	0.274
Leukopenia	18	1.6 to 209	0.021	0.6	0.1 to 5.8	0.658
Thrombocytopenia	40.8	2.0 to 856	0.018	1.1	0.2 to 6.3	0.937
Ophthalmic sicca	10.7	1.1 to 105.3	0.043	1.1	0.1 to 11.4	0.958

*OR, odds ratio; CI, coefficient interval; aPL, antiphospholipid antibodies; p value < 0.05 was considered statistical significant*.

### Sicca

The quadruple aPL positivity is also highly associated with sicca. Sicca was found in 50% of patients with quadruple positivity vs. 9% of patients with single or double aPL positivity (*p* = 0.043). Patients with triple-only aPL positivity presented a similar prevalence as those with single or double positivity (9.1 vs. 9%).

**Figure 4:**
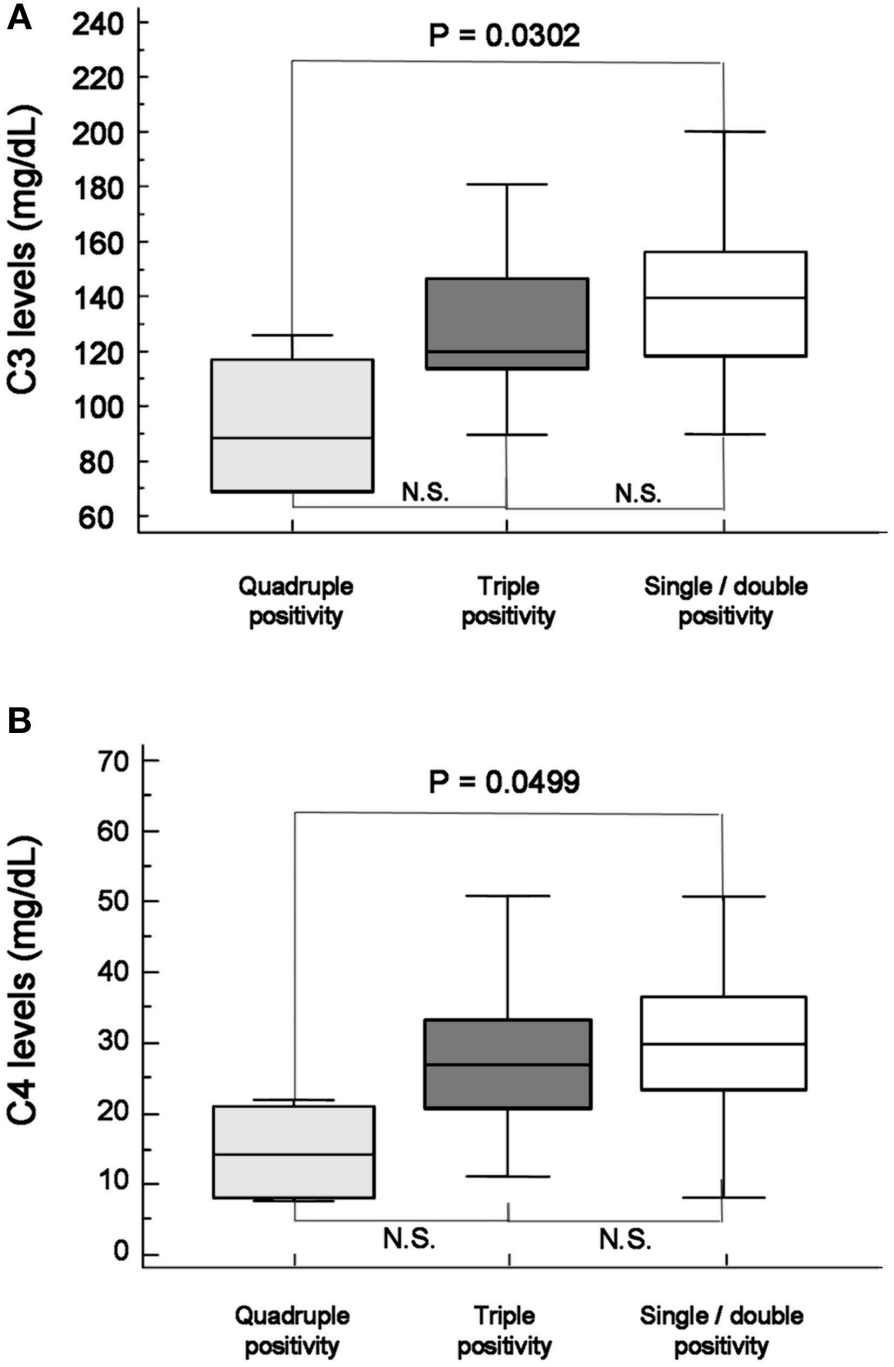
Mean levels of C3 **(A)** and C4 **(B)** complement factor in patients with sicca and quadruple aPL positivity (gray) were significantly lower than patients with sicca and single/double aPL positivity(white). When they were compared with patients with triple positivity (dark), they were also lower, but did not become significant.

Mean levels of C3 and C4 complement factors in patients with sicca and quadruple aPL positivity were significantly lower than in patients with sicca and single/double aPL positivity (Figure 4).

## Discussion

Prevalence of B2-CIC in the APS Serbian cohort is similar to that previously described for B2A-CIC in a Spanish cohort of patients with APS-clinical events (27, 28). Up to date, only the classical clinical APS manifestations (thrombosis and gestational morbidity) have been studied in patients with immune-complexes (28). For the first time, the present study has established the association between the two new biomarkers (B2G-CIC and B2M-CIC) and the clinical manifestations related to APS.

Despite the fact that B2GPI is a protein that is permanently present in the blood, most of the patients who were positive for anti-B2GPI antibodies did not form B2-CIC. The absence of B2-CIC could be explained because B2GPI circulates mainly in a “circular” conformation where the interactions between domains 1 and 5 would result in the shielding of the domain 1 epitopes (36) that would not be accessible for aPL. The CIC would form when β2GPI acquires a “hook” conformation where the epitopes are exposed (37).

The immune complexes clearance system (based on FC receptors and complement receptors) would immediately withdraw B2-CIC from circulation. B2-CIC would only be detectable in patients in whom the immune complex clearance system is insufficient to eliminate them.

This hypothesis is supported by the fact that immunocomplexes formed with IgG are more infrequent than those formed by IgM. Both immunoglobulins activate complement by classical pathway and would benefit from the immune complex clearance by complement receptors. However, B2-CIC can also be removed by the Fc gamma receptors system present in several cells of the immune system (38). This additional clearance mechanism is not present in the IgM immune complexes. Only a single receptor for IgM Fc is known and although its functions are still not completely known, they seem to be related to the regulation of the immune response and have no relation with the clearance of immune complexes (39, 40).

LR is the most frequent cutaneous manifestation in APS patients, although it is not included as classification criteria. This can be the first clinical manifestation in primary APS in up to 40% of the patients and in up to 70% in patients with SLE and APS (41). In the first description of the syndrome, G. Hughes included LR as part of APS. LR was associated with venous and arterial thrombosis, spontaneous abortions, neurological manifestations and thrombocytopenia (42). Our study has established the association between LR and CIC for the first time. At present, the pathophysiology of LR is not completely known (43, 44). CIC could be deposited in the venous territory with slow blood circulation. The deposition would cause the formation of microthrombosis, thus hindering the return circulation that would, in consequence, lead to the appearance of LR.

Dry eye syndrome (DES) is a disorder affecting the tears and ocular surface accompanied by dryness, irritation, foreign body sensation, light sensitivity, and itching. It is more prevalent in patients with autoimmune diseases (45). Humoral immunity plays an important role in the pathogenesis of DES. B cell hyperactivity is related with the presence of autoantibodies, hypergammaglobulinemia, and the clinical/serological phenotypes mediated by immune complexes. The pathogenic role of autoantibodies in DES has been demonstrated by the autoantibodies transferred (46). Sjögren's syndrome (SjS) is characterized by the combination of DES and dry mouth (xerostomia) with a progressive lymphocytic infiltration on the lachrymal and salivary glands. Autoantibodies present in serum samples of SjS patients are autoantibodies to antigens Ro(SSA), La(SSB), or both (47, 48).

DES has been previously described as a common ocular manifestation in patients with antiphospholipid syndrome (49) that had already been described in the first publications in which APS was considered as an entity independent of other systemic autoimmune diseases (50). Most of these APS patients were positive for dry Schirmer's tests but did not fulfill the criteria to be qualified as having Sjogren' syndrome (51).

The prevalence of sicca in our cohort is high (17.5%), although it is within the range of prevalence described for the adult population (5–30%) (52). However, the prevalence of sicca was significantly higher (54.5%) when only patients positive for B2-CIC were considered. All the patients with APS and sicca in our cohort were negative for anti-SSA and anti-SSB autoantibodies, Schirmer's test positive and did not suffer dry mouth, suggesting that we are facing an autoantibody-mediated non-Sjogren Dry-Eye syndrome.

Thrombocytopenia, the most common non-criteria manifestation of APS (53), was significantly more frequent in patients with B2-CIC. The association between presence of aPL CIC and thrombocytopenia has also been described for B2A-CIC (21). Platelet activation is the first step in thrombus formation (54–56). The immune-complexes are bound exclusively to the platelet thrombus (54) and this phenomenon leads to the amplification of platelets activation and aggregation mediated by platelet membrane receptors. This mechanism would explain the thrombocytopenia in patients with CIC. Leukopenia was also more frequent in patients with CIC. Leukopenia is a typical feature in SLE patients and is associated with thrombocytopenia (57–59).

Complement activation plays an important role in the pathogenesis of thrombosis induced by aPL. Lower C3 and C4 complement factors levels in patients with B2-CIC reported in this study suggest a consumption of complement. Autoantibodies within the immune complexes could activate the classical pathway of the complement cascade. The complement activation and consumption could be mediated by CIC, as has already been described in other pathologies mediated by immune-complexes (60–63).

In contrast, complement is not consumed in patients with B2A-CIC. IgG and IgM isotype antibodies can fix the complement by the classic pathway, but not IgA isotype antibodies (21).

Triple aPL positivity is an increased risk factor for thromboembolic events in APS patients (64). In this study, patients with triple aPL positivity showed a higher prevalence of pulmonary embolism and infarction, and also catastrophic APS.

In our cohort, leukopenia, livedo reticularis, sicca, and especially thrombocytopenia were more frequent in patients with quadruple aPL positivity. These findings suggest that the presence of B2-CIC would be related to the pathogenesis of livedo reticularis, thrombocytopenia, leukopenia, and sicca.

The main limitation of the study is the low number of patients and the non-inclusion of asymptomatic controls. A new confirmatory study with a higher number of individuals is mandatory. Despite the low number of APS patients, the results are very strong. As the present work is retrospective and the serum samples were obtained after the patients had suffered the thrombotic events, it was not possible to determine whether patients with quadruple positivity had a higher risk of experiencing thrombosis or cardiovascular events than the rest and whether this risk was due to triple positivity or the presence of immunocomplexes. Further prospective studies with a large number of participants to help resolve these issues are necessary.

In summary, for the first time, B2-CIC (IgG and IgM) has been described and its presence has been associated with clinical manifestations related to APS as thrombocytopenia, LR, sicca ophthalmic, and leukopenia as well as higher complement consumption.

## Author contributions

AS and LS conceived the project, designed the research, discussed the results and wrote the manuscript. LS, NS, and GB were responsible for the patients' care and clinical data collection. DP, LN, and MS performed the determinations of antiphospholipid antibodies. MS and LN made the quantification of immune complexes. AS and DP were responsible for the database and the statistical analysis. All authors contributed to the data interpretation, reviewed the manuscript and agreed with the final version.

We want to recognize the magnificent work of the European Forum on Antiphospholipid Antibodies providing opportunities for clinicians and translational scientists involved in the field of the APS to meet and strengthen collaborations.

### Conflict of interest statement

The authors declare that the research was conducted in the absence of any commercial or financial relationships that could be construed as a potential conflict of interest. The reviewer JK and the handling Editor declared their shared affiliation.

## Supplementary material

Supplementary Figure 1 Box and whiskers box of the distribution of the B2G-CIC and B2M-CIC values.

Supplementary Table 1 Complementary clinical characteristics of the 57 APS patients included in the study.

Supplementary Table 2 Comparison of the clinical characteristics of the two groups of patients. Group 1: patients with circulating immune complexes of IgG or IgM bound to beta-2-glycoprotein I (B2-CIC +), group 2: patients without circulating immune-complexes (B2-CIC –). *SEM, standard error of the mean, p-value*<*0.05 was considered statistical significant, OR, odd ratio, CI, confidence interval*.

Supplementary Table 3 Additional clinical characteristics of APS patients with triple aPL positivity vs. single or double aPL positivity.

## Abbreviations

OR: Odds ratio
CI: Confidence interval
aB2GPI: Anti-Beta-2 glycoprotein-I antibodies
aCL: Anti-cardiolipin antibodies
CIC: Circulating immune complexes
B2-CIC: Circulating immune-complexes of IgG or IgM bounded to Beta-2 glycoprotein-I.
