# Supplementary figures and images for: Presence of Immune Complexes of IgG/IgM Bound to B2-glycoprotein I Is Associated With Non-criteria Clinical Manifestations in Patients With Antiphospholipid Syndrome
Source: Front Immunol. 2018 Nov 20;9:2644. doi: 10.3389/fimmu.2018.02644 (PMC6256181; doi:10.3389/fimmu.2018.02644)

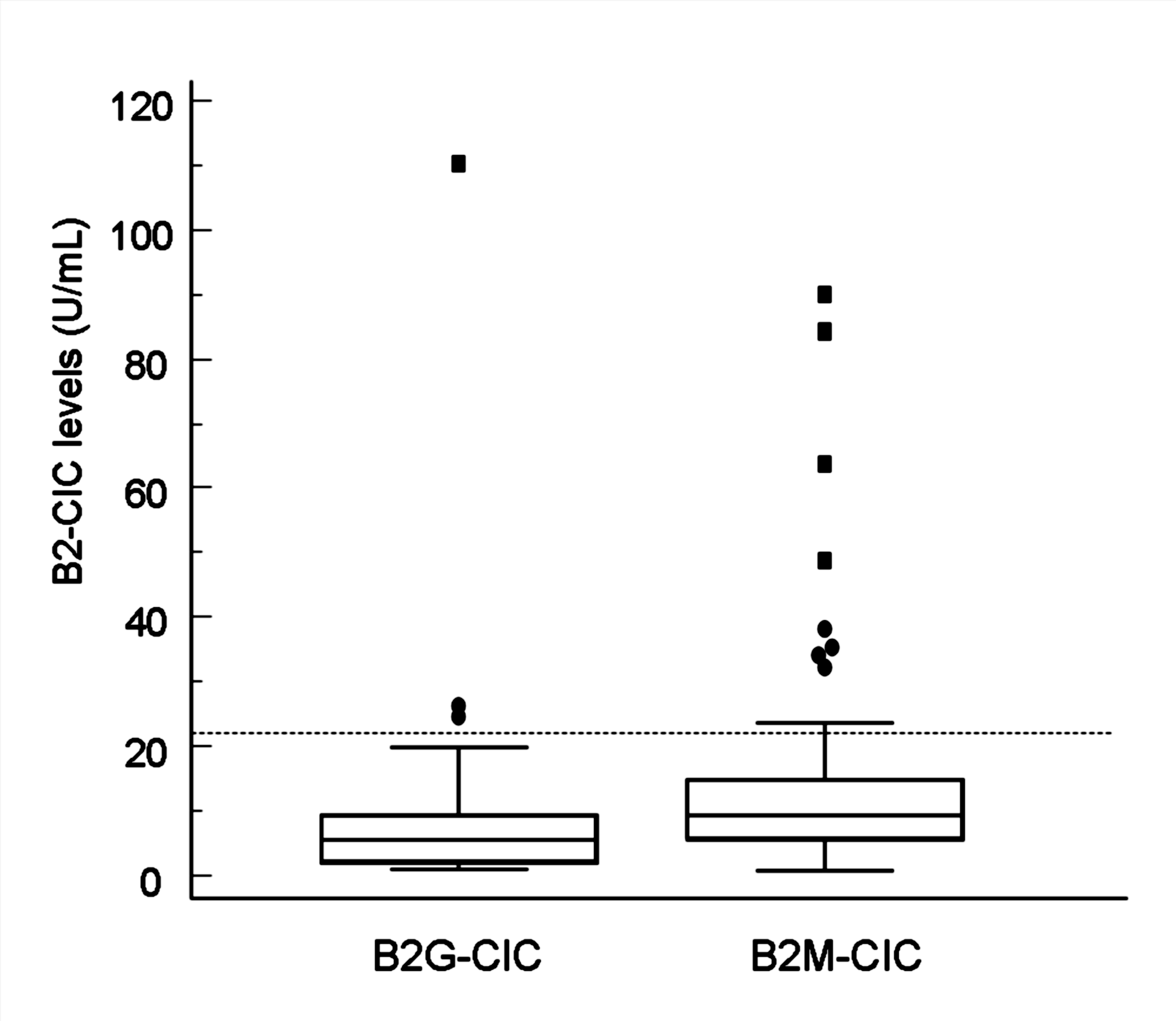

Supplement: Supplementary Figure 1 — Box and whiskers box of the distribution of the B2G-CIC and B2M-CIC values. [file Image_1.TIF]
