# Supplementary material for: Presence of Immune Complexes of IgG/IgM Bound to B2-glycoprotein I Is Associated With Non-criteria Clinical Manifestations in Patients With Antiphospholipid Syndrome
Source: Front Immunol. 2018 Nov 20;9:2644. doi: 10.3389/fimmu.2018.02644 (PMC6256181; doi:10.3389/fimmu.2018.02644)
Supplement: Supplementary Table 1 — Complementary clinical characteristics of the 57 APS patients included in the study. [file Table_1.DOC]

**Supplementary Table 1**. Complementary clinical characteristics of the 57 APS patients included in the study.

| **CONDITION** | **Number of patients** | **%** |
| --- | --- | --- |
| **Neurological diseases** |  |  |
| Transient ischemic attack | 24 | (43.9%) |
| Stroke | 22 | (38.6%) |
| Chorea | 3 | (5.3%) |
| Cerebellar ataxia | 1 | (1.8%) |
| Epilepsy | 8 | (14.0%) |
| Migraine | 10 | (17.5%) |
| Transient global amnesia | 1 | (1.8%) |
| Multi-infarct dementia | 4 | (7.0%) |
| Acute ischemic encephalopathy | 1 | (1.8%) |
| Cephalea | 17 | (29.8%) |
| Multiple sclerosis like | 2 | (3.5%) |
| Psychosis/depression | 5 | (8.8%) |
| Others neuropathies | 4 | (7.0%) |
| **Cardiovascular diseases** |  |  |
| Acute myocardial infarction | 1 | (1.8%) |
| Unstable angina | 1 | (1.8%) |
| Chronic cardiomyopathy | 1 | (1.8%) |
| Vegetations | 8 | (14.0%) |
| Pseudo infective endocarditis | 5 | (8.8%) |
| Valve thickening and dysfunction | 2 | (3.5%) |
| **Respiratory diseases** |  |  |
| Pulmonary embolism and infarction | 14 | (24.6%) |
| Primary pulmonary hypertension | 1 | (1.8%) |
| Secondary pulmonary hypertension | 3 | (5.3%) |
| Major pulmonary arterial thrombosis | 2 | (3.5%) |
| Pulmonary microthrombosis | 14 | (24.6%) |
| Pleuritis | 5 | (8.8%) |
| Other pulmonary manifestations | 4 | (7.0%) |
| **Rheumatologic diseases** |  |  |
| Avascular necrosis of bone | 1 | (1.8%) |
| Arthralgias | 18 | (31.6%) |
| Arthritis | 16 | (28.1%) |
| **Skin diseases** |  |  |
| Livedo reticularis | 18 | (31.6%) |
| Skin ulcerations | 5 | (8.8%) |
| Inferior extremity superficial thrombophlebitis | 25 | (43.9%) |
| Pseudovasculitic lesions | 9 | (15.8%) |
| Superficial cutaneous necrosis | 2 | (3.5%) |
| Digital gangrene | 2 | (3.5%) |
| Anetoderma | 1 | (1.8%) |
| Malignant atrophic papulosis like lesions | 1 | (1.8%) |
| **Other diseases** |  |  |
| Retinal artery thrombosis | 1 | (1.8%) |
| Hypothyroidism | 3 | (5.3%) |
| Optic neuropathy | 1 | (1.8%) |
| Ophthalmic sicca | 10 | (17.5%) |
| Thrombocytopenia | 14 | (24.6%) |
| Autoimmune hemolytic anemia | 2 | (3.5%) |
| Microangiopathic hemolytic anemia | 3 | (5.3%) |
| Leukopenia | 11 | (19.3%) |
| Stress | 13 | (22.8%) |
| Menopause | 20 | (35.1%) |
