# Supplementary material for: Presence of Immune Complexes of IgG/IgM Bound to B2-glycoprotein I Is Associated With Non-criteria Clinical Manifestations in Patients With Antiphospholipid Syndrome
Source: Front Immunol. 2018 Nov 20;9:2644. doi: 10.3389/fimmu.2018.02644 (PMC6256181; doi:10.3389/fimmu.2018.02644)
Supplement: Supplementary Table 2 — Comparison of the clinical characteristics of the two groups of patients. Group 1: patients with circulating immune complexes of IgG or IgM bound to beta-2-glycoprotein I (B2-CIC +), group 2: patients without circulating immune-complexes (B2-CIC –). SEM, standard error of the mean, p-value<0.05 was considered statistical significant, OR, odd ratio, CI, confidence interval. [file Table_2.DOC]

*Supplementary Table 2. Comparison of the clinical characteristics of the two groups of patients. Group 1: patients with circulating immune complexes of IgG or IgM bound to beta-2-glycoprotein I (B2-CIC +), group 2: patients without circulating immune-complexes (B2-CIC -).* SEM: standard error of the mean, p value <0.05 was considered statistical significant, OR: odd ratio, CI: confidence interval.

|  | **Group 1** | | **Group 2** | |  |
| --- | --- | --- | --- | --- | --- |
| **CONDITION** | **B2-CIC +**  **N=11** | **% /SEM** | **B2-CIC-**  **N=46** | **% / SEM** | **p value** |
| Age (years) | 49.9 | ±4.2 | 47 | ±1.7 | 0.476 |
| Sex (women) | 9 | (81.8%) | 27 | (58.7%) | 0.153 |
| Catastrophic APS | 0 | (0%) | 4 | (8.7%) | 0.577 |
| Primary APS | 5 | (45.5%) | 30 | (65.2%) | 0.227 |
| **Antiphospholipid antibodies (aPL) positive** |  |  |  |  |  |
| Anti-cardiolipin IgG antibodies | 6 | (54.5%) | 18 | (39.1%) | 0.352 |
| Anti-cardiolipin IgM antibodies | 5 | (45.5%) | 20 | (43.5%) | 0.906 |
| Anti-beta2-glycoprotein I IgG antibodies | 6 | (54.5%) | 20 | (43.5%) | 0.508 |
| Anti-beta2-glycoprotein I IgM antibodies | 7 | (63.6%) | 22 | (47.8%) | 0.346 |
| Lupus anticoagulant | 10 | (90.9%) | 28 | (60.9%) | 0.079 |
| Triple aPL positivity | 4 | (36.4%) | 11 | (23.9%) | 0.455 |
| **Other immunological markers** |  |  |  |  |  |
| Reactive protein C elevated | 3 | (27.3%) | 10 | (21.7%) | 0.700 |
| CH50 | 0 | (0%) | 1 | (2.2%) | 1.0 |
| C3 complement factor low | 1 | (9.1%) | 2 | (4.3%) | 1.0 |
| C4 complement factor low | 0 | (0%) | 1 | (2.2%) | 1.0 |
| Anti-DNA antibodies | 2 | (18.2%) | 4 | (8.7%) | 0.326 |
| Antinuclear autoantibodies | 6 | (54.5%) | 18 | (39.1%) | 0.555 |
| Rheumatoid Factor | 0 | (0%) | 2 | (4.3%) | 1.0 |
| Anti-Ro antibodies | 0 | (0%) | 2 | (4.3%) | 1.0 |
| Anti-La antibodies | 0 | (0%) | 1 | (2.2%) | 1.0 |
| **APS Pathology** |  |  |  |  |  |
| ***Gestational morbidity*** |  |  |  |  |  |
| Women in fertile age | 8 | (72.7%) | 26 | (56.5%) | 0.325 |
| Women with fetal loss | 6 | (75%) | 15 | (57.7%) | 0.642 |
| Mean fetal loss | 1.7 | ±0.30 | 1.5 | ±0.3 | 0.786 |
| Women with late fetal loss | 5 | (62.5%) | 10 | (38.5%) | 0.417 |
| Women with early fetal loss | 1 | (12.5%) | 5 | (19.2%) | 1.000 |
| ***Thrombotic events*** |  |  |  |  |  |
| Arterial thrombosis | 8 | (72.7%) | 36 | (78.3%) | 0.694 |
| Venous thrombosis | 6 | (54.5%) | 21 | (45.7%) | 0.596 |
| Pulmonary embolism | 3 | (27.3%) | 11 | (23.9%) | 0.816 |
| Inferior extremity deep vein thrombosis | 1 | (9.1%) | 11 | (23.9%) | 0.426 |
| Superior extremity arterial thrombosis | 2 | (18.2%) | 1 | (2.2%) | 0.092 |
| **Additional Pathology** |  |  |  |  |  |
| **Neurological disease** |  |  |  |  |  |
| Transient ischemic attack | 6 | (54.5%) | 19 | (41.3%) | 0.427 |
| Stroke | 5 | (45.5%) | 17 | (37%) | 0.603 |
| Cerebellar ataxia | 1 | (9.1%) | 0 | (0%) | 0.193 |
| Epilepsy | 0 | (0%) | 8 | (17.4%) | 0.332 |
| Migraine | 2 | (18.2%) | 8 | (17.4%) | 1.0 |
| Transient global amnesia | 0 | (0%) | 1 | (2.2%) | 1.0 |
| Multi-infarct dementia | 1 | (9.1%) | 3 | (6.5%) | 1.0 |
| Acute ischemic encephalopathy | 0 | (0%) | 1 | (2.2%) | 1.0 |
| Cephalea | 4 | (36.4%) | 13 | (28.3%) | 0.598 |
| Multiple sclerosis like | 1 | (9.1%) | 1 | (2.2%) | 0.263 |
| Psychosis/depression | 1 | (9.1%) | 4 | (8.7%) | 1.0 |
| Others neuropathies | 2 | (18.2%) | 2 | (4.3%) | 0.107 |
| **Cardiovascular disease** |  |  |  |  |  |
| Acute myocardial infarction | 0 | (0%) | 1 | (2.2%) | 1.0 |
| Unstable angina | 0 | (0%) | 1 | (2.2%) | 1.0 |
| Chronic cardiomyopathy | 0 | (0%) | 1 | (2.2%) | 1.0 |
| Vegetations | 1 | (9.1%) | 7 | (15.2%) | 1.0 |
| Pseudo infective endocarditis | 1 | (9.1%) | 4 | (8.7%) | 1.0 |
| **Respiratory disease** |  |  |  |  |  |
| Secondary pulmonary hypertension | 1 | (9.1%) | 2 | (4.3%) | 0.527 |
| Primary pulmonary hypertension | 1 | (9.1%) | 0 | (0%) | 0.193 |
| Major pulmonary arterial thrombosis | 1 | (9.1%) | 1 | (2.2%) | 0.263 |
| Pulmonary microthrombosis | 2 | (18.2%) | 12 | (26.1%) | 0.584 |
| Pleuritis | 0 | (0%) | 5 | (10.9%) | 0.252 |
| Other pulmonary manifestations | 2 | (18.2%) | 2 | (4.3%) | 0.107 |
| **Rheumatologic disease** |  |  |  |  |  |
| Avascular necrosis of bone | 0 | (0%) | 1 | (2.2%) | 1.0 |
| Systemic lupus erythematosus | 6 | (54.5%) | 16 | (34.8%) | 0.231 |
| Arthralgias | 4 | (36.4%) | 14 | (30.4%) | 0.704 |
| Arthritis | 4 | (36.4%) | 12 | (26.1%) | 0.496 |
| **Skin disease** |  |  |  |  |  |
| Pseudovasculitic lesions | 1 | (9.1%) | 8 | (17.4%) | 0.673 |
| Superficial cutaneous necrosis | 1 | (9.1%) | 1 | (2.2%) | 0.351 |
| Skin ulcerations | 1 | (9.1%) | 4 | (8.7%) | 1.0 |
| Inferior extremity superficial thrombophlebitis | 5 | (45.5%) | 20 | (43.5%) | 0.906 |
| Digital gangrene | 1 | (9.1%) | 1 | (2.2%) | 0.351 |
| Malignant atrophic papulosis like lesions | 0 | (0%) | 1 | (2.2%) | 1.0 |
| Anetoderma | 0 | (0%) | 1 | (2.2%) | 1.0 |
| **Other diseases** |  |  |  |  |  |
| Retinal artery thrombosis | 0 | (0%) | 1 | (2.2%) | 1.0 |
| Hypothyroidism | 0 | (0%) | 3 | (6.5%) | 0.384 |
| Optic neuropathy | 0 | (0%) | 1 | (2.2%) | 1.0 |
| Microangiopathic hemolytic anemia | 0 | (0%) | 3 | (6.5%) | 1.0 |
