# Supplementary material for: Presence of Immune Complexes of IgG/IgM Bound to B2-glycoprotein I Is Associated With Non-criteria Clinical Manifestations in Patients With Antiphospholipid Syndrome
Source: Front Immunol. 2018 Nov 20;9:2644. doi: 10.3389/fimmu.2018.02644 (PMC6256181; doi:10.3389/fimmu.2018.02644)
Supplement: Supplementary Table 3 — Additional clinical characteristics of APS patients with triple aPL positivity vs. single or double aPL positivity. [file Table_3.doc]

**Supplementary Table 3.** Additional clinical characteristics of APS patients with triple aPL positivity vs single or double aPL positivity.

| **CONDITION** | **Triple**  **positivity N=15** | **%** | **Single / double**  **positivity**  **N=42** | **%** | **p value** |
| --- | --- | --- | --- | --- | --- |
| **Neurological diseases** |  |  |  |  |  |
| Transient ischemic attack | 7 | (46.7%) | 18 | (42.9%) | 0.799 |
| Stroke | 3 | (20%) | 19 | (45.2%) | 0.085 |
| Chorea | 1 | (6.7%) | 2 | (4.8%) | 0.777 |
| Cerebellar ataxia | 1 | (6.7%) | 0 | (0%) | 0.263 |
| Epilepsy | 4 | (26.7%) | 4 | (9.5%) | 0.101 |
| Migraine | 3 | (20%) | 7 | (16.7%) | 0.771 |
| Transient global amnesia | 0 | (0%) | 1 | (2.4%) | 0.547 |
| Multi-infarct dementia | 1 | (6.7%) | 3 | (7.1%) | 0.951 |
| Acute ischemic encephalopathy | 1 | (6.7%) | 0 | (0%) | 0.263 |
| Cephalea | 4 | (26.7%) | 13 | (31%) | 0.756 |
| Multiple sclerosis like | 1 | (6.7%) | 1 | (2.4%) | 0.439 |
| Psychosis/depression | 1 | (6.7%) | 4 | (9.5%) | 0.737 |
| Others neuropathies | 1 | (6.7%) | 3 | (7.1%) | 0.951 |
| **Cardiovascular diseases** |  |  |  |  |  |
| Acute myocardial infarction | 1 | (6.7%) | 0 | (0%) | 0.263 |
| Unstable angina | 0 | (0%) | 1 | (2.4%) | 0.547 |
| Chronic cardiomyopathy | 0 | (0%) | 1 | (2.4%) | 0.547 |
| Vegetations | 2 | (13.3%) | 6 | (14.3%) | 0.927 |
| Pseudo infective endocarditis | 1 | (6.7%) | 4 | (9.5%) | 0.737 |
| Valve thickening and dysfunction | 2 | (13.3%) | 0 | (0%) | 0.066 |
| **Respiratory diseases** |  |  |  |  |  |
| Primary pulmonary hypertension | 0 | (0%) | 1 | (2.4%) | 1.000 |
| Secondary pulmonary hypertension | 1 | (6.7%) | 2 | (4.8%) | 0.777 |
| Major pulmonary arterial thrombosis | 1 | (6.7%) | 1 | (2.4%) | 0.439 |
| Pulmonary microthrombosis | 6 | (40%) | 8 | (19%) | 0.106 |
| Pleuritis | 0 | (0%) | 5 | (11.9%) | 0.311 |
| Other pulmonary manifestations | 2 | (13.3%) | 2 | (4.8%) | 0.265 |
| **Rheumatologic diseases** |  |  |  |  |  |
| Avascular necrosis of bone | 0 | (0%) | 1 | (2.4%) | 1.000 |
| Systemic lupus erythematosus | 8 | (53.3%) | 14 | (33.3%) | 0.176 |
| Arthralgias | 4 | (26.7%) | 14 | (33.3%) | 0.634 |
| Arthritis | 2 | (13.3%) | 14 | (33.3%) | 0.139 |
| **Skin diseases** |  |  |  |  |  |
| Livedo reticularis | 7 | (46.7%) | 11 | (26.2%) | 0.143 |
| Skin ulcerations | 2 | (13.3%) | 3 | (7.1%) | 0.467 |
| Inferior extremity superficial thrombophlebitis | 8 | (53.3%) | 17 | (40.5%) | 0.389 |
| Pseudovasculitic lesions | 3 | (20%) | 6 | (14.3%) | 0.602 |
| Superficial cutaneous necrosis | 2 | (13.3%) | 0 | (0%) | 0.066 |
| Digital gangrene | 2 | (13.3%) | 0 | (0%) | 0.066 |
| Anetoderma | 0 | (0%) | 1 | (2.4%) | 1.000 |
| **Other diseases** |  |  |  |  |  |
| Retinal artery thrombosis | 1 | (6.7%) | 0 | (0%) | 0.263 |
| Hypothyroidism | 1 | (6.7%) | 2 | (4.8%) | 0.777 |
| Optic neuropathy | 0 | (0%) | 1 | (2.4%) | 1.000 |
| Ophthalmic sicca | 3 | (20%) | 7 | (16.7%) | 0.771 |
| Thrombocytopenia | 6 | (40%) | 8 | (19%) | 0.106 |
| Autoimmune hemolytic anemia | 2 | (13.3%) | 0 | (0%) | 0.066 |
| Microangiopathic hemolytic anemia | 0 | (0%) | 3 | (7.1%) | 0.288 |
| Leukopenia | 4 | (26.7%) | 7 | (16.7%) | 0.400 |
| Stress | 5 | (33.3%) | 8 | (19%) | 0.259 |
| Menopause | 4 | (26.7%) | 16 | (38.1%) | 0.426 |
